# Interpreting time-series COVID data: reasoning biases, risk perception, and support for public health measures

**DOI:** 10.1038/s41598-021-95134-z

**Published:** 2021-08-02

**Authors:** Jason L. Harman, Justin M. Weinhardt, James W. Beck, Ivy Mai

**Affiliations:** 1 Department of Psychology, Louisiana State University, Baton Rouge, USA; 2 Haskayne School of Business, University of Calgary, Calgary, Canada; 3 Department of Psychology, University of Waterloo, Waterloo, Canada

**Keywords:** Psychology, Human behaviour

## Abstract

Effective risk communication during the COVID-19 pandemic is critical for encouraging appropriate public health behaviors. One way that the public is informed about COVID-19 numbers is through reports of daily new cases. However, presenting daily cases has the potential to lead to a dynamic reasoning bias that stems from intuitive misunderstandings of accumulation. Previous work in system dynamics shows that even highly educated individuals with training in science and math misunderstand basic concepts of accumulation. In the context of COVID-19, relying on the single cue of daily new cases can lead to relaxed attitudes about the risk of COVID-19 when daily new cases begin to decline. This situation is at the very point when risk is highest because even though daily new cases have declined, the active number of cases are highest because they have been accumulating over time. In an experiment with young adults from the USA and Canada (N = 551), we confirm that individuals fail to understand accumulation regarding COVID-19, have less concern regarding COVID-19, and decrease endorsement for public health measures as new cases decline but when active cases are at the highest point. Moreover, we experimentally manipulate different dynamic data visualizations and show that presenting data highlighting active cases and minimizing new cases led to increased concern and increased endorsement for COVID-19 health measures compared to a control condition highlighting daily cases. These results hold regardless of country, political affiliation, and individual differences in decision making. This study has implications for communicating the risks of contracting COVID-19 and future public health issues.

## Introduction

Mitigating COVID-19 depends heavily on actions taken by individuals, from mask-wearing and physical distancing to getting vaccinated^1–3^. How these decisions are informed depend on both the accuracy of information available as well as the accuracy of individuals’ interpretation of that information. Across the world individuals are presented with an abundance of information regarding COVID-19 including: daily new cases, number of deaths, new health recommendations, new government mandates, and more recently vaccination information. Though the reliability of COVID information has improved from earlier stages of the pandemic^4^ and important advances have been made understanding how people evaluate misinformation^5–7^, understanding how people (mis)interpret accurate information remains a needed area of research^8^. In this paper we investigate whether a perception bias common in dynamic reasoning tasks translate to COVID-19 decisions, and we test possible debiasing techniques in the form of alternative information presentation standards.

There is a large literature indicating that individuals, regardless of training and education, can be poor judges of risk, particularly when interpreting health related numbers and statistics^9–11^. Even medical experts misunderstand risk information. For example, physicians at a continuing education course about breast cancer screenings were provided with information on base rates, sensitivity, and specificity in standard probability format. They were then asked what the chances are a patient has breast cancer with a positive test on the screening. Choosing between four alternatives, only 21% of the physicians gave the correct response which is lower than if they would have guessed randomly^9^. Several subsequent studies have demonstrated that giving the same information in a natural frequency format (i.e., 10 out of 1,000 women have breast cancer) instead of as a probability statement dramatically improves performance^9^.Building on this work we propose that the commonly used visualization of presenting new daily COVID-19 cases can lead to a reduction in concerns about COVID-19 and support for public health behaviors at precisely the time when these behaviors are most needed. COVID-19 cases vary over time within a given geographic location, making adherence to protective health behaviors more important at certain times, relative to others. Taken as a whole, this means it is critically important for individuals to have access to valid information about COVID-19 cases in their area, and more so, to be able to accurately interpret this information to understand the importance of exhibiting precautionary behaviors at a given point in time. Fortunately, reliable information about COVID-19 cases is readily available to the population through the Center for Disease Control and Prevention^12,13^, the World Health Organization, and local government websites. Although individuals have access to valid information regarding COVID-19, there are reasons to believe that individuals may often misinterpret this valid information, thereby forming inaccurate perceptions of the risks of contracting COVID-19^14^. In particular, we propose that individuals misunderstand the dynamic nature of COVID-19, which leads to biased perceptions of the risk of COVID-19.

## COVID-19 as a dynamic system

Dynamic systems at their most basic are characterized by stocks and flows. A stock is a variable that accumulates over time. Conversely, a flow is a variable that either increases (an inflow) or decreases the stock (an outflow). The classic example of such a system is a bathtub, in which the current water level (stock) is determined by the water entering the tub from the tap (inflow), minus the water exiting the tub from the drain (outflow). The stock variable accumulates over time as a function of both the inflow and the outflow. COVID-19 can be viewed as a dynamic system because the number of *active cases* in an area is the stock, which is accumulating as a function of *new daily cases* that are the inflow, and *resolved cases* (i.e., recoveries + deaths) that are the outflow.

Unfortunately, there is a great deal of evidence indicating that individuals, even those with advanced training in math and science, have a poor understanding of the behavior of dynamic systems^15,16^. For instance, individuals routinely misjudge the effects of changes in calorie intake on weight^17^. Similarly, many adults misjudge the influence that reducing carbon emissions will have on the concentration of greenhouse gasses in the atmosphere^18^. The most common explanation for failing to understand these dynamic stock-and-flow systems is that individuals use the correlation heuristic^15^. The correlation heuristic is a bias in which individuals erroneously assume that the inflow of a system (e.g., calories eaten, carbon emissions) is positively correlated with its stock (e.g., weight, total CO_2_ in the atmosphere). However, individuals tend to ignore accumulation in the stock relative to its’ outflow (e.g., calories burned, carbon removal). For example, to reduce atmospheric CO_2_ (stock), carbon removal (outflow) must be greater than carbon emissions (inflow). This work highlights the problems individuals have understanding dynamic phenomenon that have profound consequences on their lives. Building on this literature we propose that individuals are likely to misunderstand the dynamic nature of COVID-19 when information about the inflow is emphasized rather than the stock.

When accessing the WHO, CDC websites^12,13^ or even searching COVID-19 data on Google the first displays of data focus on new and total cases rather than information on active cases. However, emphasizing information about new cases (inflow) will lead individuals to misinterpret the risk of COVID-19 because of the correlation heuristic. Although, the number of new cases and the number of active cases is positively correlated, there will be a time where new cases decline but active cases are still increasing. Because each new case requires several weeks to be resolved, there is a critical period during which new cases are in decline (i.e., the “curve is flattening”), yet active cases are at the highest point. As such, this critical period represents the point of highest actual risk (highest number of infectious people in an area), yet it is also likely to be coupled with declining perceptions of risk because of the correlation heuristic. It is during this time that individuals may be inclined to “ease up” on precautionary behaviors, even though this is the period when those behaviors are most critical for stopping the spread of the virus. Therefore, we argue that endorsement for public health measures may be undermined by a failure to correctly understand the accumulation of active cases.

## Current study

We conducted an experiment in both the United States and Canada to test whether time series presentations of daily new COVID-19 cases biases concern ratings for COVID-19 and endorsement of public health behaviors. Moreover, we tested three different data visualization formats of COVID-19 data to test if presenting active cases reduces this bias. These visualizations varied how the number of active cases were presented either numerically, graphically, or both. The third visualization that combined both the numerical and the graphical display was designed to minimize the salience of new cases and emphasize active cases to make correlation heuristic judgments consistent with actual risk. These were compared to the control condition that only presented new and resolved cases. We assessed the degree to which these different visualization styles affected concern ratings and endorsement of public health behaviors against the spread of COVID-19.

Participants included young adults residing in both the United States and Canada. Finally, we also measured individual differences in political affiliation, decision making style, and general risk perceptions regarding COVID-19. Political affiliation has been related to differences in perceptions of the risk of COVID-19 and support for public health measures^19^. Analytical decision making has small effects in dynamic systems errors^20^ and has been correlated with discerning between COVID-19 accurate information and misinformation regarding COVID-19^7^. General risk perception for COVID-19 could influence sensitivity and variance to changing case numbers.

Our two main hypotheses are:

### Hypothesis 1

Consistent with use of the correlation heuristic, endorsement of public health behaviors and concern ratings will decrease when new cases decline but when active cases are at the highest level.

### Hypothesis 2

Data visualizations emphasizing active cases will reduce the discrepancy between endorsement of public health behaviors and concern ratings and actual risk based on total active cases.

## Experiment

**Figure 1:**
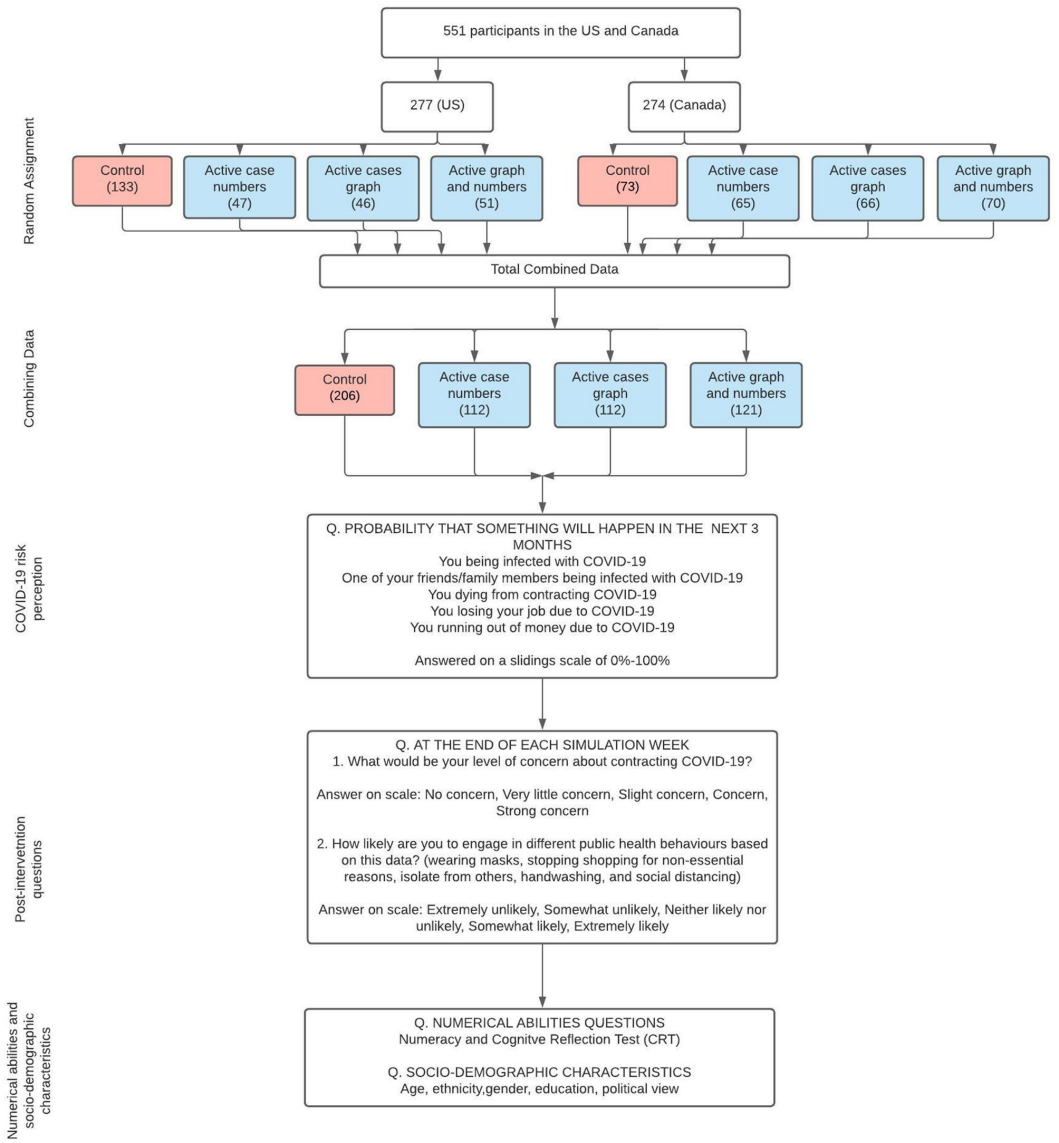
Experimental procedure.

**Figure 2:**
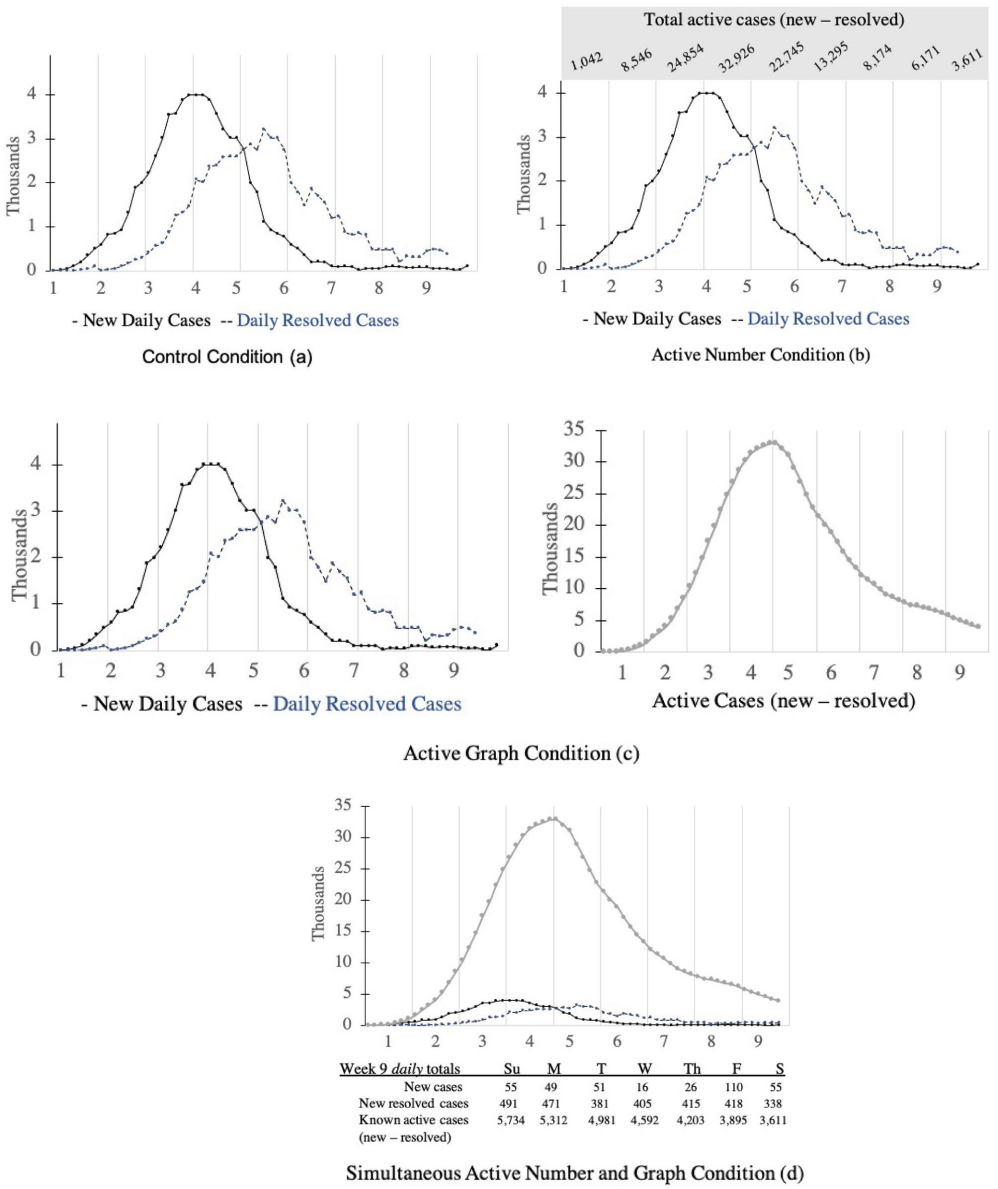
(**a**–**d**) Visual display of experimental stimuli that participants evaluated. (**a**) Control condition: participants were shown an unfolding graph of new daily cases (black line) and daily resolved cases (blue line). (**b**) Active numbers condition: participants were shown the same unfolding graph as condition and they were also provided with unfolding calculated total active case numbers for each week at the top of the graph. (**c**) Active graph condition: participants were shown two side by side graphs, they were shown the same unfolding graph as condition (**a**) and an unfolding graph of the total active case numbers for each week. (**d**) Simultaneous Active Numbers and Graph Condition: Participants were shown an unfolding graph of new daily cases (black line), new resolved cases (blue line), total active cases (grey line) together as well as a table break down of case numbers for each week.

For this study, a total of 551 participants from the USA and Canada completed an experiment and questionnaire (Fig. 1) in which they were shown simulated data summarizing COVID-19 cases in a given location. Specifically, participants were shown data across nine experimental trials, wherein each trial represented 1 week (Fig. 2). During each simulated week new data were added to the graph and participants were told to imagine the data were representative of COVID-19 cases in their city. Following each simulated week, participants indicated how concerned they would be about contracting COVID-19, given the data they had been shown up to that point. Participants also reported their likelihood of engaging in various public health behaviors; specifically: wearing masks, avoiding non-essential shopping, isolating from others, handwashing, and social distancing. Individuals indicated their political beliefs, and general risk perceptions of COVID-19. We also measured analytical decision making style using a test of numeracy^21^ and the cognitive reflection test (CRT)^22^. Numeracy and the CRT have been shown to be reliable predictors of decision making ability across multiple tasks^23^.

The simulation was designed such that new cases increased each week until week 3, at which point new cases were at their highest level. Following week 3, new cases declined for the remainder of the simulation. However, the highest number of active cases occurred at week 4. Therefore, we were interested in participant responses between week 3 (the highest number of new cases) and week 4 (the highest number of active cases). Although week 4 is associated with the highest levels of risk, we predicted that during week 4 participants would actually report less concern and lower protective behavioral intentions relative to week 3.

We designed three visualizations where the estimated number of active cases are presented, eliminating the need to subtract resolved cases from new cases to determine risk. Participants were randomly assigned to see one of these four conditions. Participants in the *control condition* only saw the information in Fig. 2a. In the *active number condition* (Fig. 2b), participants were presented with the same graph as the control condition, but the number of active cases was presented to them at the top of the graph numerically. In the *active graph condition* (Fig. 2c), participants were also shown the same graph as the control condition, yet participants in this condition were also shown an additional graph of the active cases. Thus, the “active number” and “active graph” conditions contained the same information, yet this information was either displayed numerically or graphically, depending on the condition. These two conditions eliminate the need for participants to perform any calculations to determine the number of active cases, testing whether simple availability of information would improve risk perceptions. Finally, in the *simultaneous active graph and number condition* (Fig. 2d), participants saw the numbers for new cases, resolved cases, and active cases and they also saw active cases visually depicted on the same graph as the new and resolved cases. This condition simultaneously provides all relevant data while also minimizing the saliency of number of new cases and makes the saliency of active cases most apparent by illustrating the magnitude of accumulation.

## Results

**Figure 3:**
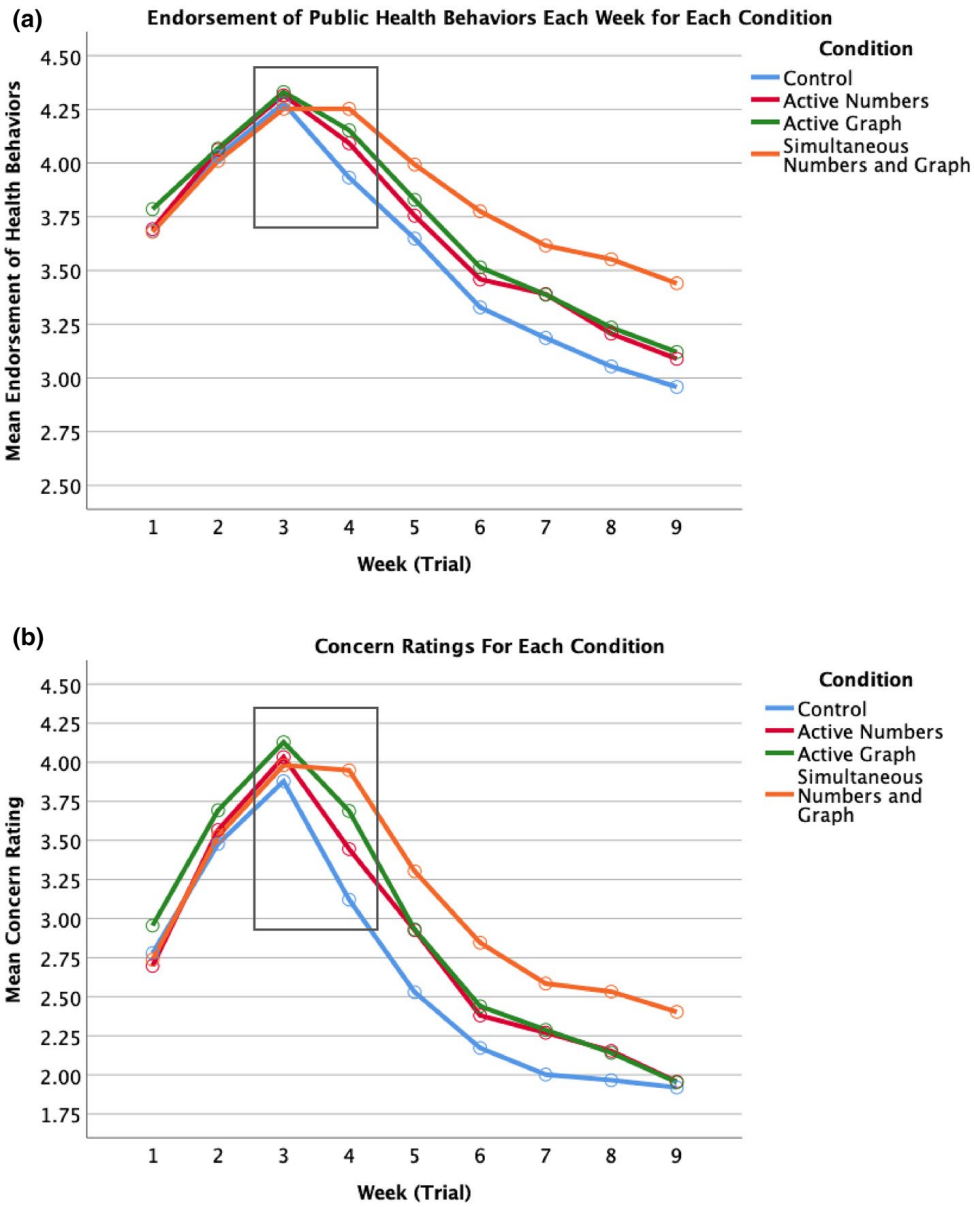
(**a**) Endorsement of public health behaviors each week by condition. (**b**) Concern ratings each week by condition.

Across our analyses we found no evidence of between country differences. Therefore, we collapsed the data across countries. Table 1 shows the correlation between the individual differences measured in the study and the average concern ratings and average endorsement of public health behaviors across the simulation. We used repeated measures ANOVA to test whether condition had moderating effect on endorsement of behaviors across the simulated 9 weeks. Controlling for political beliefs, general risk perceptions of COVID-19, and decision making style there was a significant interaction between time and condition for endorsement of public health behaviors, *F*(24, 4352) = 6.80, *p* < 0.001 Partial eta^2^ = 0.04 (Fig. 3a). The same pattern of results holds for concern over contracting COVID-19 *F*(24, 4136) = 7.49, *p* < 0.001 Part eta^2^ = 0.04 (Fig. 3b). Significance of results does not change when no controls are used. To test whether time series presentation of COVID data leads to the predicted bias, we analyzed responses from week 3 and week 4. The highlighted data points in Fig. 3 show that both health behaviors and concern decrease from week 3 to week 4 for each condition but for those in the *simultaneous active graph and number condition*.

**Table 1:** Descriptive and correlation table.

Variable	M	SD	Country	Political beliefs	Numeracy and CRT	Risk perception of COVID-19	Avg. concern ratings	Avg. endorsement of public health behaviors
Country	0.5	0.5	1	0.04**	0.42**	− 0.02	0.04**	0.02
Political Beliefs	4.72	2.16	0.04**	1	0.02	− 0.03	− 0.15**	− 0.22**
Analytical Decision Making Style	5.6	2.15	0.42**	0.02	1	− 0.07**	− 0.05**	− 0.07**
General Risk Perception of COVID-19	32.91	16.8	− 0.02	− 0.03	− 0.07**	1	0.12**	0.02
Avg. Concern Ratings	2.84	1.24	0.04**	− 0.15**	− 0.05**	0.12**	1	0.71**
Avg. Endorsement of Public Health Behaviors	3.68	0.93	0.02	− 0.22**	− 0.07**	0.02	0.71**	1

N = 551. Country (0 = USA, 1 = Canada), Political Beliefs (1 = Very Liberal, 9 = Very Conservative), Analytical Decision Making Style measured using numeracy and CRT, General Risk Perceptions of COVID-19 (see “Method” section), Avg. Concern rating is the average across all concern ratings in the experiment, Avg. Endorsement of Public Health Behaviors is the average across all endorsement of public health behaviors in the experiment.

**p<.01

### Hypothesis 1: Concern and endorsement of public health behaviors decrease as active COVID cases peak

**Figure 4:**
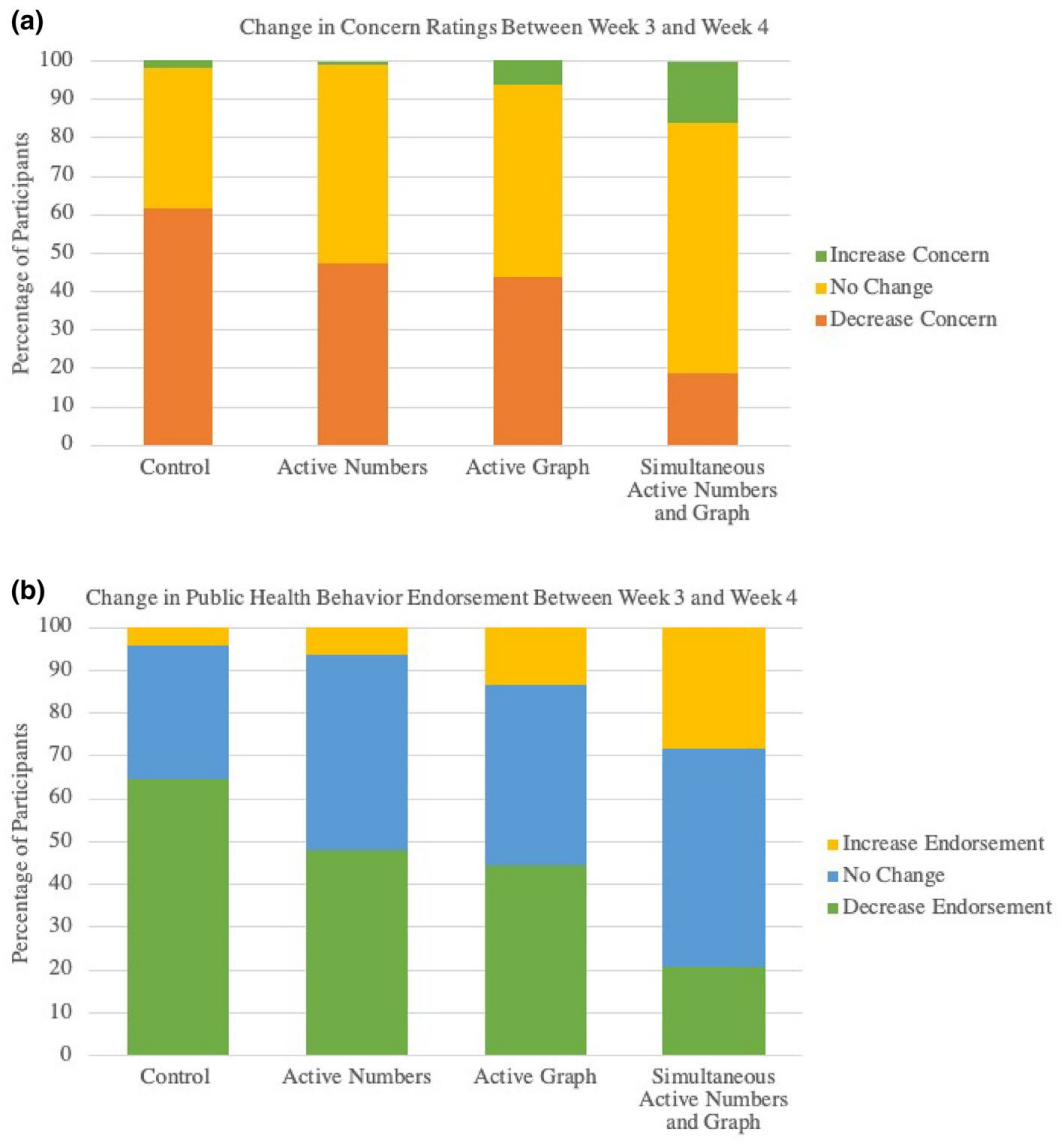
(**a**) Perecent of participants who changed concern ratings between week 3 and week 4. (**b**) Perecent of participants who changed endorsement for public health behaviors between week 3 and week 4.

In the control condition, there was a decrease in the endorsement of public health behaviors between Week 3 (M = 4.29, SD = 0.69) and Week 4 (M = 3.94, SD = 0.74), *t*(205) = 11.86, *p* < 0.001, (Cohen’s *d* =  − 0.49). The same pattern holds for concern, decreasing between Week 3 (M = 3.88, SD = 1.05) and Week 4 (M = 3.14, SD = 1.03), *t*(202) = 14.08, *p* < 0.001, (Cohen’s *d* = 0.71). Figure 4 graphs the percentage of participants whose concern and health behavior ratings decreased, increased, or stayed the same between weeks 3 and 4. In the control condition the majority of participants (> 60%) decreased their ratings between the two key time points.

### Hypothesis 1: Testing the correlation heuristic as a mechanism

To directly test that the correlation heuristic is used when interpreting dynamic COVID-19 data, we correlated concern ratings and endorsement of health behaviors provided after each simulated week with both new cases (correlation heuristic) and active cases (actual risk). Next, we tested the difference between these two correlations. If participants relied on the correlation heuristic when providing these ratings, then a stronger correlation is expected between new cases and concern ratings and endorsement of health behaviors, relative to the correlation between active cases and concern ratings and endorsement of health behaviors. Thus, in the control condition and the first two visualizations, we expected new cases to be a stronger predictor, relative to active cases. Yet, we expected this pattern to be reversed for those in the *simultaneous active graph and number condition*, where the most salient cue to inform use of the correlation heuristic is active cases.

These results are summarized in Table 2. Consistent with our predictions, in the control condition, both concern (z =  − 13.882, *p* < 0.001) and health behaviors (z =  − 8.313, *p* < 0.001) were more strongly influenced by new cases, relative to active cases. This same pattern held in both the *active numbers* and *active graph conditions*; that is, participants continued to be more strongly influenced by information regarding new cases, relative to active cases. However, for participants in the *simultaneous active graph and number condition*, new cases and active cases were equally influential on concern ratings (z =  − 0.803, *p* = 0.211). Nonetheless, participants in this condition continued to be more strongly influenced by new cases, compared to active cases (z =  − 2.607, *p* = 0.005), though the absolute difference in correlations was far smaller in this condition relative to the control condition. Taken as a whole, these results indicate the *simultaneous active numbers and graph condition* was effective for reducing reliance on the correlation heuristic; the other manipulations did not meaningfully reduce reliance on this heuristic.

**Table 2:** Dependent correlation between active cases and new cases with health behaviors and concern ratings by condition.

Dependent correlation with endorsement of health behaviors			Dependent correlation with concern ratings		
Condition	Dependent correlation	Significance test for comparison	Condition	Dependent correlation	Significance test for comparison
Control	Correlation with health behaviors	z = − 8.313, *p* < 0.001	Control	Correlation with concern ratings	z = − 13.882, *p* < 0.001
Active	0.269		Active	0.274	
New	0.402		New	0.491	
Active number	Correlation with Health Behaviors	z = − 4.914, *p* < 0.001	Active number	Correlation with concern ratings	z = − 7.896, *p* < 0.001
Active	0.299		Active	0.352	
New	0.405		New	0.515	
Active graph	Correlation with health behaviors	z = − 4.014, *p* < 0.001	Active graph	Correlation with concern ratings	z = − 8.463, *p* < 0.001
Active	0.272		Active	0.364	
New	0.36		New	0.536	
Simultaneous active graph and number	Correlation with health behaviors	z = − 0.803, *p* = 0.211	Simultaneous active graph and number	Correlation with concern ratings	z = − 2.607, *p* = 0.005
Active	0.302		Active	0.412	
New	0.319		New	0.464	

### Hypothesis 2: Highlighting active cases while minimizing new cases eliminates inconsistent perceptions

The simultaneous *active numbers and graph condition* assumes the use of the correlation heuristic and displays data in a way that the correlation heuristic will produce consistent risk perceptions by making active cases more salient through showing graphically that active cases are substantially larger than new cases because of accumulation. Over all weeks, participants in the *active numbers and graph condition* had higher endorsement of public health behaviors (Fig. 3a) (M = 3.85, SD = 0.69) than the control condition (M = 3.58, SD = 0.73) (Cohen’s *d* = 0.38) but was not significantly different than the other visualizations. For concern ratings (Fig. 3b), participants *active numbers and graph condition* (M = 3.13, SD = 0.86) had higher concern than those in the control condition (M = 2.68, SD = 0.85; Cohen’s *d* = 0.53), *active numbers condition* (M = 2.82, SD = 0.85; Cohen’s *d* = 0.36) and was marginally higher than those in the *active graph condition* (M = 2.88, SD = 0.82; *p* = 0.058; Cohen’s *d* = 0.30).

Looking at the key time point between week 3 and week 4, only those in the *simultaneous active numbers and graph* condition did not decrease endorsement of public health behaviors on average from weeks 3 to 4 (Week 3: M = 4.27, SD = 0.67; Week 4: M = 4.27, SD = 0.69, Cohen’s *d* = 0.01). We found similar results for concern ratings for those in the *simultaneous active numbers and graph condition* (Week 3: M = 4.03, SD = 1.01; Week 4: M = 4.00, SD = 1.02, Cohen’s *d* = 0.03). As can be seen in Fig. 4a, participants in the *simultaneous active numbers and graph condition* had the highest percentage of participants increasing scores in Week 4 compared to Week 3 for both endorsement of health behaviors (*X*^2^ (6, 551) = 79.90, *p* < 0.0001) and concern (*X*^2^ (6, 544) = 75.091, *p* < 0.0001).

Those in the *active numbers condition* and *active graph condition* both decreased endorsement of public health behaviors and lowered their concern ratings between week 3 and week 4. Those in the *active numbers condition* decreased endorsement of public health behaviors between Week 3 (M = 4.31, SD = 0.63) and Week 4 (M = 4.089, SD = 0.71), *t*(112) = 6.21, *p* < 0.001, (Cohen’s *d* = 0.33). Similarly, concern ratings decreased between Week 3 (M = 3.99, SD = 1.05) and Week 4 (M = 3.40, SD = 1.13), *t*(112) = 8.41, *p* < 0.001, (Cohen’s *d* = 0.54).

Those in the *active graph condition* decreased endorsement of public health behaviors between Week 3 (M = 4.29, SD = 0.80) and Week 4 (M = 4.12, SD = 0.85), *t*(112) = 4.78, *p* < 0.001, (Cohen’s *d* = 0.21). Similarly, concern ratings decreased between Week 3 (M = 4.07, SD = 1.07) and Week 4 (M = 3.63, SD = 1.1), *t*(112) = 6.54, *p* < 0.001, (Cohen’s *d* = 0.41).

## Discussion

The global COVID-19 pandemic has magnified the importance of accurate and useful scientific communication. With every corner of the planet affected in some way, even small errors in interpreting valid information could have large real-world effects. In this paper we have focused on one of the more ubiquitous forms of risk communication, numbers of new daily cases. Reported frequently in television and print news, time series graphs of new daily cases similar to those used in this study are the first result when googling “covid numbers.” There are potential advantages to this form of data presentation. A large body of research has shown that natural frequencies are more intuitively and accurately processed than probabilities generally and more specifically in medical contexts^24–26^. On the other hand, research has also highlighted potential biases in understanding the basic processes in dynamic accumulation, leading to misunderstandings of stocks and flows, such as weight and atmospheric CO_2_.

Our experimental data demonstrates that changes in concern over COVID-19 and willingness to comply with protective behaviors corresponded with new daily cases. Most of the time daily cases are commensurate with actual risk, meaning individuals are likely to behave appropriately given the risks they face. However, relying on new daily cases to assess risk and determine one’s behavior will lead individuals astray in one important situation. Specifically, immediately following a peak in new cases, individuals are likely to misjudge the risks associated with contracting COVID-19. In this situation, we found concern and intention to engage in protective behaviors reduced with the drop in new cases, even though this is the time when actual risk is at its highest because active cases are at their highest point. Our results are consistent with this bias risk perception resulting from the use of the correlation heuristic which has been hypothesized to lead to stock flow failures in other domains and have been shown to be resistant to debiasing strategies, education, or training^15,20^.

We designed three visualizations to overcome the stock flow bias. The first two visualizations present the total number of active cases either numerically or graphically, eliminating the need to calculate the difference between new and resolved cases. However, we found that these visualizations did not eliminate the stock flow bias. Despite knowing the true number of active cases, most participants still seem to base their concern and willingness to comply with health behaviors on the number of new cases. This result is likely due to new cases being a salient piece of information, both visually and through familiarity as it is a commonly reported COVID-19 number. The *simultaneous active numbers and graph* visualization was designed to both display the number of active cases while simultaneously minimizing the saliency of new cases. This visualization successfully decreased the bias found in the other conditions. We believe the most important feature of the visualization is that the graph of active cases was presented on the same scale as the new and resolved cases. This both highlighted the magnitude of active cases while also de-emphasized new cases, making active cases the most salient cue likely to be used in the correlation heuristic. This condition also led to overall greater endorsement of public health behaviors and concern with COVID-19. Moreover, these results were found after controlling for political orientation, general COVID-19 risk perceptions, and decision-making style.

Important implications from our results include advice for both the communication of COVID infection data, timing of public policy measures, and how to effectively communicate vaccination information moving forward. In terms of communicating COVID infection information, individuals charged with communicating this information should be aware of the correlation heuristic and importantly the stock flow bias. Presenting and highlighting the most direct correlate of risk (estimated active cases) may be preferable in some circumstances. For both scientists and public policy makers, understanding that following a peak in new cases when numbers begin to fall might be the most dangerous time because of the combination of higher active cases and laxed vigilance by the public. Both communicating this increased risk and refraining from the temptation of loosening restrictions too early are important prescriptions from the current results. Finally, as vaccines become increasingly available in some parts of the world the need for informed data communication will persist and take new forms. Understanding how these dynamic numbers are interpreted is as important as presenting the most valid information. Valid information misunderstood, can have unintended behavioral consequences.

## Limitations

Our sample consisted of young adults at two universities in U.S. and Canada. Though the use of only western college students does limit generalizability, and future studies need to include a more diverse sample, university students are a key population for COVID-19 mitigation and even results that are only shown in this population could be consequential. Although young adults are at less risk of mortality from COVID-19, they have an outsized impact on transmission^27,28^ with many universities debating mandatory vaccination for students^29^. Beyond the importance of the specific population we used in this study, the robustness of stock flow failures in previous studies suggest that the current results will generalize across age, education, and culture^15,20,30,31^. An additional limitation of the current study is the lack of a manipulation check. Verifying basic understandings of graphs or the information provided in the visualizations could account for additional variance in the data. A third limitation to the current study is the use of simulated data as opposed to real time data. We used simulated data to keep stimuli consistent between countries, creating the simulated data to match the actual trends experienced in each geographic location. Data was collected in the fall semester of 2020. During data collection at the Louisiana State University, COVID infections were at a plateau between a second and third wave of COVID infections^32^ while at The University of Calgary (Canada) COVID infections were at a plateau prior to their first major wave^33^. We based the simulated data on the data from Alberta for the sample in Canada and Louisiana for the sample run in the USA. Finally, the visualizations we tested add information to the data presentation. Although we found that participants understood the figures, it is possible that adding complexity to data visualizations could reduce their efficacy^34,35^.

## Method

This study was approved by the Louisiana State University Ethics Board as well as the University of Calgary Research Ethics Board. Participants gave informed consent to be in the study and the study was performed in accordance with all relevant guidelines and regulations.

### Participants

Participants were students enrolled at the University of Calgary and Louisiana State University and were given course credit for participation. A total of 628 university students participated in our study, 77 dropped out of the study or were removed for failing attention checks, leaving a total sample of 551. Of 551 students, 277 are American and 274 are Canadian. For our American sample, 19.5% identified as male, 67.9% identified as White/European descent, and the average age was 20.07 (*SD* = 3.03). For our Canadian sample, 50.4% identified as male, 45.6% indicated that they were White/European descent, and the average age was 20.70 (*SD* = 2.87).

### Experimental manipulation

Participants were randomly assigned to one of four conditions: Control Condition, Active Numbers Condition, Active Graph Condition, and Simultaneous Numbers and Graph Condition. An initial programming error led to more American participants being assigned to the control condition (*N* = 133). Figure 2 contains a visual depiction of the stimuli presented in each of the four conditions. Participants in the *Control Condition* were presented with a graph that plotted only new and resolved cases. In this condition, the participant would have to calculate active cases themselves. This condition is the most similar to current media presentations of COVID-19 data in the two locals. Participants in the *Active Number Condition* were presented with a graph in which new and resolved cases were plotted and the calculated number of active cases were displayed at the top of the graph. Participants in the *Active Graph Condition* were presented with two graphs. On the left new and resolved cases were displayed (same as control condition), and on the right active cases were plotted. Finally, participants in the *Simultaneous Numbers and Graph* Condition were presented with new cases, resolved cases, and active cases visually on the same graph and were also presented with the number of active cases. This condition combines the previous two conditions.

### Procedure

Participants were first asked to fill out a COVID-19 risk perception measure. Participants were presented with an unfolding graph showing COVID-19 of daily new cases and daily new resolved cases over the course of 10 weeks. Participants were told that the data were simulated and were not a true representation of the current COVID-19 case numbers in their area. For the American sample, numbers were simulated to match with the actual peak number of new cases in Louisiana during the summer of 2020. For the Canadian sample, the simulated data from the American stimuli were divided by 10 which roughly matched the case counts in Alberta during the summer of 2020. Data were presented 9 different times, each time with a new week being revealed. At each time point, participants were asked to indicate their level of concern about contracting COVID-19 based on that week’s data and were also asked to indicate the likelihood of engaging in public health behaviors (see below). Following the repeated trials, participants completed measures of numeracy, cognitive reflection, political orientation, and demographic questions.

## Measures

### General COVID-19 risk perception

Participants were first provided with 5 different COVID-19 related scenarios and were asked to indicate the probability (0–100) of them happening in the next 3 months. These involved scenarios such as the participants or their friends/family being infected with the virus, dying from the virus, losing their job, or running out of money due to COVID-19. There were no differences between American and Canadian participants.

### Concern ratings

After each trial of simulated data all participants were asked: “Based on the current data you have seen above, what would be your level of concern about contracting COVID-19?” Participants rated their concern on a five-point Likert-type scale from (1) “No Concern” to (5) “Strong Concern”.

### Endorsement of public health behaviors

After each trial of simulated data all participants were asked to indicate the likelihood they would engage in five different public health behaviors: “Wear a face mask in public”, “Go out shopping or eating for non-essential reasons” (reverse coded), “Wash/sanitize your hands before entering and exiting public spaces and business”, “Socially distance at least 6ft”, “Stay at home and isolate from others outside your immediate family”. Participants rated their endorsement of the public health behaviors on a five-point Likert-type scale (1) “Extremely Unlikely” to (5) “Extremely Likely” for each health behavior. These ratings were then averaged for each trial.

### Political affiliation

Participants were asked to identify their political view on a 9-point Likert-type scale ranging from (1) “Extremely Liberal” to (9) “Extremely Conservative”.

### Decision making style

Participants completed the Rasch-based Numeracy Scale^21^ and Frederick’s^22^ Cognitive Reflection Test. The combination of these two measures as an indicator of decision-making ability across multiple tasks^23^.
